# Asymptomatic COVID-19 as a Risk Factor of Diabetic Ketoacidosis and Mucormycosis: A Case Report and Review of the Literature

**DOI:** 10.1155/2021/2765867

**Published:** 2021-11-11

**Authors:** Soheila Torabiyan, Behnam Dalfardi, Mojgan Sanjari

**Affiliations:** Endocrinology and Metabolism Research Center, Institute of Basic and Clinical Physiology Sciences, Kerman University of Medical Sciences, Kerman, Iran

## Abstract

Mucormycosis is a lethal and life-threatening fungal infection. Several cases describing the association of COVID-19 and mucormycosis have been reported. In this article, we report a 58-year-old female with a history of diabetes mellitus type 2 who presented by diabetic ketoacidosis, rhino-orbital mucormycosis, and COVID-19. The patient was treated with liposomal amphotericin B and debridement of necrotic tissue of the rhino-orbital area and paranasal sinuses. Unfortunately, she passed away a few days after orbital surgery. We also conducted a review of the literature and reported 3 other similar cases that suffered from mucormycosis in association with COVID-19 and diabetic ketoacidosis and discussed the importance of this association.

## 1. Introduction

Mucormycosis is an invasive and fatal fungal infection that is caused by Mucorales, an order of zygomycete fungi [1, 2]. This infection is very rare and opportunistic and most commonly seen in underlying compromising conditions such as diabetes mellitus (DM), extensive corticosteroid use, iron overload, hematologic malignancies, neutropenia, primary immunodeficiency, and treatment with immunosuppressants [1–3]. The most common forms of involvement are rhino-orbital-cerebral, pulmonary, and cutaneous [1]. Based on the site of infection and the patient's underlying predisposing condition, the all-cause mortality rate of mucormycosis may reach up to about 80%, particularly in patients with central nervous system involvement [2].

Here, we describe a type 2 DM patient who was involved with diabetic ketoacidosis (DKA), rhino-orbital mucormycosis, and COVID-19 to emphasize the point that development of opportunistic bacterial and fungal infections is a considerable event in patients with SARS-CoV-2 infection and can affect their length of hospital stay and outcome.

## 2. Case Presentation

A 58-year-old woman presented to the emergency department with a 4-day history of right-sided face swelling, nausea, and intractable vomiting since 2 days before hospitalization. Her past medical history included hypertension and poorly controlled insulin-dependent type 2 DM. On admission, physical examination showed a body temperature of 37.5°c (orally), pulse rate of 90 beats/minute, respiratory rate of 25 breaths/minute, blood pressure of 140/90 mmHg, and a room-air arterial oxygen saturation of 94% (by finger pulse-oximetry). In addition, unilateral swelling, erythema, and facial tenderness in the right side together with right eye proptosis were detected. The patient's right eye had no light perception and no pupillary light reflex. Neurologic examination was also suggestive of the involvement of the 3^rd^, 4^th^, and 6^th^ cranial nerves. On admission, laboratory investigations resulted in the following values: white blood cell count (×10³/*µ*l): 15; creatinine (mg/dl): 2.1; blood sugar (mg/d): 350; hemoglobin A1C (%): 8.4; C-reactive protein (mg/l): 36; urine ketone: 3+; and venous blood gas study: PH: 7.22; HCO3 (mmol/l): 11.7; PCO2 (mmHg): 27.

According to the patient's clinical scenario and the presence of hyperglycemia and metabolic acidosis, she was diagnosed as a case of diabetic ketoacidosis, and treatment with intravenous fluid and insulin was started.

**Figure 1:**
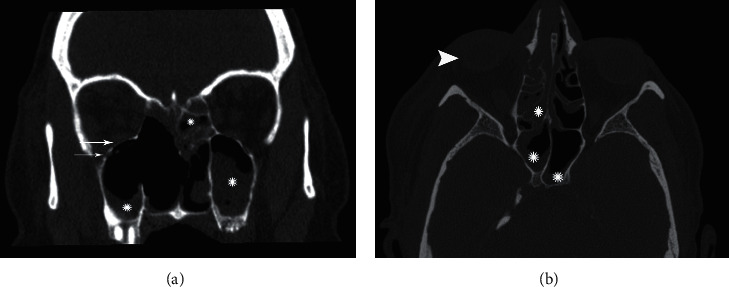
(a) Destruction of orbital bone (white arrows) and sinus involvement (white asterisk). Destruction of the nasal septum is also seen. (b) Proptosis secondary to eye involvement by mucor (white arrowhead) and sinus involvement (white asterisk).

Regarding the possibility of sinusitis, she underwent a noncontrast paranasal sinus and orbit computed tomographic scan (CT) (Figure 1). CT imaging revealed mucosal thickening of all paranasal sinuses, erosive bone lesions involving right-side lamina papyracea, middle and inferior turbinates, and right-side ethmoidal air-cells. All the findings were suggestive of erosive fungal sinusitis (Figure 1). For this reason, she was prepared for endoscopic sinus surgery.

**Figure 2:**
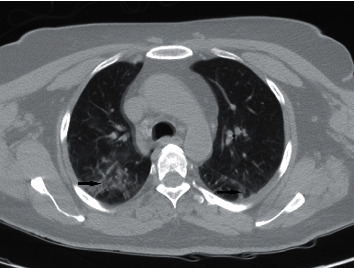
Noncontrast thoracic CT scan (axial view) showing bilateral lung infiltrations (black arrows).

Although the patient had no clinical manifestation, a screen SARS-Cov-2 PCR assay (nasopharyngeal swab test) was performed before surgery that yielded a positive result. We repeated this test for confirmation which was positive again. Noncontrast imaging of the chest was performed (Figure 2).

**Figure 3:**
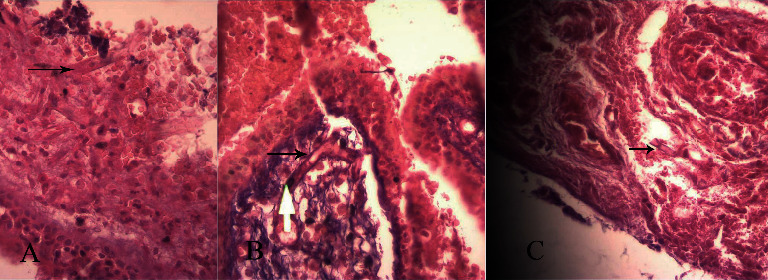
(a–c) Tissue biopsy of sinonasal tissue showing infiltration of inflammatory cells and broad-based fungal hyphae (black arrows), diagnostic for mucomycosis.

The patient underwent endoscopic sinus evaluation and surgery. The findings were the presence of necrotic tissue in right-side nasal cavity and paranasal sinuses, necrosis of soft palate, and involvement of the orbital wall. Therefore, debridement of necrotic tissues was carried out. Histopathological evaluation of tissue samples confirmed mucormycosis (Figure 3). Treatment with amphotericin B liposomal (6 mg/kg/day) was started, hyperglycemia was managed, and orbital exenteration surgery was performed. The patient's SARS-Cov-2 infection was asymptomatic, and her oxygen saturation was normal; therefore, according to the available guidelines, she did not receive any specific treatment for this infection.

Despite complete surgical debridement, antifungal therapy, and aggressive management of DKA, the patient showed no treatment response and unfortunately passed away two days after surgery.

Of note, informed consent was obtained from the patient's next of kin. This work was approved by the Ethical Committee of Kerman University of Medical Sciences (Code: IR.KMU.REC.1400.207).

## 3. Discussion

We presented a case of DKA and destructive sinusitis due to mucormycosis, accompanied by an asymptomatic SARS-CoV-2 infection.

More than one year passed from the emergence of COVID-19, and yet, there is no definitive and specific treatment against it [3]. Glucocorticoids and probably remdesivir are the only drugs that have efficacy in COVID-19. Glucocorticoids are inexpensive drugs and reduce mortality in hypoxemic COVID-19 patients [4, 5]. The immune suppression caused by glucocorticoids can increase the risk of different opportunistic fungal infections in cases with COVID-19. The use of other immunomodulatory drugs such as tocilizumab and immune dysregulation induced by SARS-CoV-2 virus are the other causes [4–7].

Invasive fungal infections have been reported increasingly in COVID-19 patients. We identified more than 60 cases of COVID-19 in association with mucormycosis. Mucormycosis is an invasive and life-threatening fungal infection [1]. Mucorales require iron for growth, must evade host phagocytic defense mechanisms, and access the vasculature to disseminate. In the hyperglycemic state of DKA, phagocytic activities are disturbed. In addition, due to impaired transferrin binding in hyperglycemic acidosis, free serum iron increases. Mucorales have a ketone reductase system that allows them to thrive in hyperglycemic and acidotic conditions, a capacity that may the higher incidence of mucormycosis in patients with diabetic ketoacidosis [8].

In our literature review, we found that, in more than 85% of these cases, mucormycosis infection occurred after COVID-19 treatment with glucocorticoids. But, only in our case and three other cases, patients had association of COVID-19 and mucormycosis in the absence of taking any treatment, including glucocorticoids, for COVID-19 (Table 1) [9–11]. In the absence of receiving any treatment for COVID-19, occurrence of mucormycosis may be due to immune dysregulation caused by SARS-CoV-2 virus or immunodeficiency related to DM that was aggravated by COVID-19 [5].

DM is a common comorbidity with COVID-19. This combination increases the possibility of complications of DM (including acute complications such as DKA), risk of secondary infections (such as mucormycosis), and patients mortality [12]. DKA is a potential lethal acute complication of DM that occurs as a result of insulin deficiency and production of ketone bodies [12, 13]. Insulin deficiency occurs more often in DM type 1 [12]. Our literature review showed several cases of DKA that were precipitated by COVID-19. Some of them reported no prior history of DM [13], and some others had a history of type 2 DM [12]. The serum level of interleukin 6 (IL6) increases in both DKA and COVID-19 and can be used as a prognostic factor [13, 14]. The interaction between COVID-19 and the rennin-angiotensin-aldosterone system (RAAS) might be another mechanism that induces DKA in COVID-19 patients [13, 15]. Angiotensin-converting enzyme 2 (ACE2) is an important and necessary enzyme in RAAS that catalyzes angiotensin type 2 to angiotensin type 1 and is highly expressed in the pancreas and lungs. It works as an entry point for COVID-19, and after endocytosis of the SARS-CoV-2 virus complex, the expression of ACE2 is reduced [13, 15, 16].

Two mechanisms may cause insulin deficiency in patients with COVID-19. First, direct invasion of SARS-CoV-2 virus may cause pancreatic damage [17]. On the other hand, reduced expression of ACE2 after virus entry can prevent insulin secretion [13, 18]. These two factors might have a role in worsening of pancreatic beta cell function and the presence of DKA in our patient.

Of the four cases reported thus far (including the index case), two cases were from the United States, one was from Mexico, and the other one (index case) was from Iran. The median age was 38.5 (24 to 58) years. Two of the cases had previously diagnosed DM, but in two other cases, DM was previously undiagnosed. Three of the cases were female, and only one was male. Acute respiratory distress syndrome (ARDS) due to COVID-19 was present in only one case. DKA was present in all of these cases. Three cases died, and one case survived.

In summary, infection with SARS-CoV-2 virus may play a triggering role for predisposing patients to DKA, particularly due to its impact on pancreatic tissue. COVID-19 and its treatment regimens may finally predispose patients to mucormycosis. Physicians should consider COVID-19 and its therapies not only as a risk factor of DKA but also for concomitant mucormycosis infection.

## Figures and Tables

**Table 1:** Summary of reports on the four cases with COVID-19, DKA, and mucormycosis.

Author	Age in years/sex	Comorbid illness	Clinical presentation	Treatment for COVID-19	Treatment for mucormycosis	Organ involved by mucormycosis	Outcome
Werthman Ehrenreih/USA [9]	33/female	HTN; asthma; undiagnosed DM	Altered mentation; proptosis; DKA; and rhino-orbital mucormycosis	Remdesivir; convalescent plasma	Amphotericin B; DKA management	Rhino-orbito-cerebral mucormycosis	Died

Waizel Haiat et al./Mexico city [10]	24/female	Obesity	Rhino-orbital mucormycosis; DKA; COVID-19; and ARDS	No treatment	Amphotericin B; mechanical ventilation; DKA management; Imipenem; and linezolid	Rhino-orbital mucormycosis	Died

Alekseyev et al./USA [11]	41/male	DM type 1	COVID-19 pneumonia; DKA; and rhinocerebral mucormycosis	Hydroxy chloroquine; steroid	Amphotericin B; cefepime; DKA management; and surgical debridement	Rhino-orbito-cerebral mucormycosis	Alive

Torabian et al./Iran (index case)	58/female	DM type 2; HTN	Rhino-orbital mucormycosis DKA COVID-19 infection	No treatment	Liposomal amphotericin B; debridement of necrotic tissue; and DKA management	Rhino-orbital mucormycosis	Died

## Data Availability

The data used to support the findings of this study are included within the article.
